# Pros and Cons of the Cannabinoid System in Cancer: Focus on Hematological Malignancies

**DOI:** 10.3390/molecules26133866

**Published:** 2021-06-24

**Authors:** Natasha Irrera, Alessandra Bitto, Emanuela Sant’Antonio, Rita Lauro, Caterina Musolino, Alessandro Allegra

**Affiliations:** 1 Department of Clinical and Experimental Medicine, University of Messina, 98125 Messina, Italy; natasha.irrera@unime.it (N.I.); alessandra.bitto@unime.it (A.B.); irritalauro@gmail.com (R.L.); 2 Hematology, Azienda USL Toscana Nord Ovest, 55100 Lucca, Italy; santantonioemanuela@gmail.com; 3 Department of Human Pathology in Adulthood and Childhood, University of Messina, 98125 Messina, Italy; cmusolino@unime.it

**Keywords:** hematological malignancies, cannabinoids, medical cannabis, anti-tumor effects, leukemia, lymphoma, bone marrow transplantation

## Abstract

The endocannabinoid system (ECS) is a composite cell-signaling system that allows endogenous cannabinoid ligands to control cell functions through the interaction with cannabinoid receptors. Modifications of the ECS might contribute to the pathogenesis of different diseases, including cancers. However, the use of these compounds as antitumor agents remains debatable. Pre-clinical experimental studies have shown that cannabinoids (CBs) might be effective for the treatment of hematological malignancies, such as leukemia and lymphoma. Specifically, CBs may activate programmed cell death mechanisms, thus blocking cancer cell growth, and may modulate both autophagy and angiogenesis. Therefore, CBs may have significant anti-tumor effects in hematologic diseases and may synergistically act with chemotherapeutic agents, possibly also reducing chemoresistance. Moreover, targeting ECS might be considered as a novel approach for the management of graft versus host disease, thus reducing some symptoms such as anorexia, cachexia, fatigue, anxiety, depression, and neuropathic pain. The aim of the present review is to collect the state of the art of CBs effects on hematological tumors, thus focusing on the essential topics that might be useful before moving into the clinical practice.

## 1. Introduction

*Cannabis sativa* (*C. sativa*) has been used for centuries for different reasons, and it is the most used illegal plant. *CS* is the main source of cannabinoids (CBs). Marijuana (a greenish-gray mix of seeds, leaves, stems, and flowers of *CS*), hash, and hash oil are the main types of cannabis, but cannabis comprises more than 500 different substances [1,2]. CBs are generally cataloged as plant CBs or phytocannabinoids, endocannabinoids, and synthetic CBs [3], and both natural and synthetic CBs are believed to have neuroactive effects. To date, over 100 phytocannabinoids have been extracted, and the substances found in the greatest concentrations are acids: cannabidiolic acid, cannabinolic acid, cannabigerolic acid, cannabichromenic acid, and cannabinodiolic acid [4,5].

Delta 9–tetrahydrocannabinol (D9-THC) and cannabidiol (CBD) are the most studied compounds. THC is the main psychoactive compound contained in marijuana, and it is quickly absorbed at the pulmonary level, binds with its specific receptors in the central nervous system, and exerts its mechanism of action.

Endocannabinoids are the main part of the endocannabinoid system (ES), which includes CB receptors (CBRs) and enzymes implicated in their synthesis, delivery, and metabolization [6]. Studies performed during the last 10 years have demonstrated the possible existence of a high degree of redundancy for both the molecular targets and metabolic routes as well as the corresponding enzymes of the endocannabinoids. Numerous N-acyl-ethanolamines, monoacylglycerols, *N*-acyl amino acids, and *N*-acyldopamines/taurines/serotonines were proposed to be part of this system. These endocannabinoid-like molecules may stimulate other molecular targets independently from cannabinoid receptors. All these findings together with the discovery of new endocannabinoid-like molecules have led authors to expand the classical view of the CBs system and to look at it as the ‘endocannabinoidome’ [7,8]. The most studied endocannabinoids are *N-*arachidonoylethanolamine (AEA, or anandamide) and 2-arachidonoylglycerol (2-AG), but other compounds such as *N*-arachidonoyl dopamine, virodhamine, and 2-arachidonoylglyceryl ether (noladin ether) are also recognized as endocannabinoids [9].

Nowadays, cannabinoids may have a synthetic origin following chemical production and are mainly CB receptor ligands; they are generally used in the research field to study their mechanism of action and their function, though they have also been employed for therapeutic and recreational purposes [10]. About 140 substances have been synthesized and are used as cannabinoids, such as SR144528, WIN 55,212-2, JWH-018, UR-144, HU-210, HU-331, and JWH-133; they may be catalogued into four different families: aminoalkylindoles, classical CBs, non-classical CBs, and fatty acid amides [11,12].

It was first believed that the CBs physiological mechanism of action was due to non-specific relations with the cellular membrane; however, both in vitro and in vivo experimental models showed that the CBs mechanism of action was related to the specific binding with the CB receptors, CB1 and CB2. CB1R is widely distributed within the central nervous system (CNS), but it has also been detected in the peripheral nerve endings and in different tissues such as lung, spleen, stomach, adipose tissue, vascular endothelium, liver, urinary bladder, and prostate [6]. CB2Rs are mainly expressed on the immune cells, though they have also been found in the CNS [13] and are involved in immunomodulation and inflammatory response [14]. However, after the detection of CB1Rs and CB2Rs, their endogenous ligands were also recognized [15,16,17,18,19,20,21].

As reported above, some endocannabinoids, such as 2-AG and AEA, may bind other transmembrane proteins, comprising orphan G protein-coupled receptor 55 (GPR55), peroxisome proliferator-activated receptors (PPARs), and transient receptor potential vanilloid (TRPV) channel type 1 (TRPV1), as well as CB1Rs and CB2Rs [22,23].

Several cannabinoid receptor agonists bind more or less to CB1Rs and CB2Rs, such as classical THC and HU-210, nonclassical (CP55940), aminoalkylindole, which has a higher affinity to CB2R more than CB1R, and antagonist-inverse agonists (SR141716A) for CB1 and SR144528 for CB2. In general, many antagonists show high selectivity toward the CB1R, which allows differentiation between CB1R and CB2R, while numerous agonists show low selectivity between cannabinoid receptors. Despite this, some agonists, such as arachidonyl-2′-chlorethylamide (ACEA) compound, show CB1R high selectivity. In addition, allosteric modulators that bind to different sites other than the ligand orthosteric site affect the stimulus of cannabinoid receptor by either enhancing or reducing its activity [24].

The purpose of the present review is to provide an overview of the effects of cannabinoids in cancers, with a focus on hematological diseases. Both pre-clinical and clinical studies will be reported, and we will highlight the hypothesis that cannabinoids might be used in the field of cancer therapy (direct administration in the neoplastic mass or application of nanotechnologies) and for the management of some symptoms observed in cancers, even if their use in clinical practice needs to be carefully investigated.

## 2. Adverse Effects of Marijuana Intake: CBs and Solid Cancers

The frequency of marijuana smoking is rising and is second only to tobacco [25]. Owing to its psychoactive actions, including the abusive potential, cannabis and its derivatives fall into the category of “controlled drugs”, and their possession is prohibited in several countries. Until recent years, despite being acknowledged as a therapeutic agent, cannabis received small consideration from researchers, probably because of its psychoactive activity—which makes it a controlled drug—and its side effects [26].

A correct evaluation of the adverse effects related to marijuana use is difficult to execute due to its prohibited condition in several countries, different smoking practices, and its briefer time of exposure with respect to tobacco [27]. However, several lung complications of marijuana smoking have been reported, and short-term exposure has been correlated to dyspnea, bronchitis, coughing, pneumothorax, apical lung bullae, and pneumomediastinum [28,29,30,31,32] (Table 1).

Regarding the adverse effects on lungs in hematological patients, Khwaja et al. described two men with acute myeloid leukemia (AML) with miliary nodular lung configurations due to relevant marijuana abuse [39]. They also described two subjects with acute lymphocytic leukemia (ALL) who had a history of smoking marijuana and then presented lung opacities consistent with mold infection. Moreover, several findings sustain the hypothesis that marijuana smoking may represent a risk factor for aero tract tumors. Marijuana smoke may contain many of the same carcinogens as tobacco smoke, comprising nitrosamines, different polycyclic aromatic hydrocarbons (PAH), vinyl chlorides, phenols, and reactive oxygen species (ROS) [40,41]. Furthermore, Benzo[a]pyrene, is also present in marijuana tar, even at a greater level than in tobacco tar [40]. With respect to tobacco smoking, marijuana smoking may provoke inhalation of about three times the quantity of tar and the retaining of one-third more of the tar in the lung [42,43]. Finally, assessment of bronchial mucosal biopsy specimens from marijuana smokers without any clinically evident symptomatology shows more alterations than that reported for non–marijuana smokers in biomarkers of altered proliferation and genetic instability, such as Ki-67, DNA ploidy, and epidermal growth factor receptor [44].

Marijuana use is quite widespread. One study evaluated about 50,000 Swedish military personnel who used marijuana during the years 1969 and 1970. Subjects were traced until 2009 for lung tumor outcomes in nationally linked medical registries. At the baseline evaluation, 5,156 military stated lifetime use of marijuana, and 831 stated use of more than 50 times, defined as “heavy” use. Statistical analyses found that such “heavy” cannabis use was considerably correlated to more than a twofold risk (hazard ratio 2.12) of developing lung tumors over the 40-year follow-up period, even after correction for baseline alcohol or tobacco use and socioeconomic status [45]. Marijuana smoking has also been recognized as a causal element in other traditionally tobacco-related cancers, such as head, neck, and transitional cell carcinoma. For instance, a previous study showed a correlation between head and neck tumors in young subjects and marijuana consumption [46], while a different report that included 52 patients with transitional cell carcinoma demonstrated that 88.5% of patients had a history of regular marijuana use, while only 69.2% of the healthy controls had the same custom, with a statistically significant difference [47].

However, the negative effects of marijuana are not only restricted to the respiratory tract; the real incidence of arrhythmias is substantially underreported given the prohibition of cannabis use. Reported arrhythmias include sinus tachycardia, sinus bradycardia, and second-degree atrioventricular blockage. Marijuana smoking can contribute to acute myocardial infarction by causing coronary artery vasospasm [33] or may be an important risk factor for atrial fibrillation [34] and hepatitis [35].

Moreover, marijuana intake may affect skin and trigger Raynaud syndrome, thus causing arteritis [48]. Long-lasting, intense cannabis use has also been associated with cognitive alterations, with a significant reduction in attention and memory damage [36,37].

Among the negative effects related to CBs intake, the immune system might be affected, with the consequence of a compromised immune response against infectious diseases and tumors (anti-neoplastic immunosurveillance reduction). Continuous consumption of marijuana may also be responsible for alveolar macrophage injury, while cannabis may alter the delivery of cytokines, possibly causing the incapacity of the immune system to oppose to infections [49,50]. In this regard, marijuana can also become infected with various fungi such as *Aspergillus*, Mucorales, and *Fusarium* [51]. In an experimental study, marijuana’s mold amount was evaluated with respect to that of tobacco. A total of 100,000 colony-forming units of mold were found on marijuana vs. 200 colony-forming units on tobacco [52].

Finally, chronic cannabis intake increases the risk of cannabis dependence, which is related to several negative psychosocial outcomes such as cognitive deficiency, psychoses and depressive and anxiety alterations, poor educational outcomes, and antisocial behavior [38,53,54].

In the past years, several experimental studies have assessed the clinical efficacy of cannabis and CBs in preclinical and clinical cancer models, establishing that CBs are able to halt tumor cell growth and progression [55,56]. Remarkably, some other reports have shown that CBs can display a carcinogenic potential in specific conditions (Table 2).

Exposure of mice to Delta9-THC stimulated tumor proliferation and invasion, thus affecting the anticancer immune response, probably as a consequence of an opposite effect played by CB2R with respect to CB1R. In fact, Delta9-THC increased IL-4 and IL-10, indicating that Delta9-THC exposure may particularly inhibit the cell-mediated Th1 response by augmenting Th2-associated cytokines [60]. Similar results were obtained in two different animal lung cancer models: THC administration caused a faster proliferation of cancer implants compared to the treatment with diluent alone. The immune inhibitory cytokines IL-10 and TGF-β were increased, while IFN-γ was reduced both in the tumor site and in spleens of THC-treated mice. When anti-IL-10- or anti-TGF-β-neutralizing Abs were administered, they prevented the THC-induced increase of cancer proliferation. Antigen-presenting cells and T cells from THC-treated animals demonstrated inadequate aptitudes to produce alloreactivity. Moreover, lymphocytes from THC-treated mice transmitted the effect to normal animals, causing an enhanced tumor proliferation similar to that reported in the THC-treated mice [61]. Finally, in an in vitro study, Hart et al. reported that anandamide, THC, HU-210, and Win55,212-2 stimulated mitogenic kinase signaling in tumor cells. Treatment of the glioblastoma cell line U373-MG and the lung carcinoma cell line NCI-H292 with THC caused an augmented growth that was totally reliant on metalloproteases and epidermal growth factor receptor activity [62].

Both CB1Rs and CB2Rs were reported to be expressed in different types of cancer cells. Interestingly, both receptors were often unrevealed at the place of the tumors’ origin before neoplastic transformation [63]. Furthermore, augmented amounts of endocannabinoid have been reported in different tumors, such as prostate cancer, glioblastoma, colon cancer, hepatocellular carcinoma, skin cancer, endometrial sarcoma, pituitary adenoma, and meningioma [63,64], and the number of enzymes implicated in the endocannabinoid degradation is frequently correlated with the tumor severity. Wang et al. reported that CB1R had a tumor-suppressive activity in a genetically changed animal experimental model of a colon tumor [65]. On the other hand, CB1R is increased in hepatocellular carcinoma, and the amount of increase was associated with tumor severity in epithelial ovarian cancer [66,67,68]. In the same way, CB2R has also been demonstrated to be increased in both gliomas and HER2+ breast tumors [69,70]. Finally, it was demonstrated that CB1R and CB2R increase was associated with bad outcomes in stage IV colorectal carcinoma patients [71,72].

With regard to epidemiological studies, two diverse analyses, both retrospective and based on the population of Kaiser Permanente subscribers in Northern California, evaluated marijuana as a risk factor for tumor onset [57,73].

The correlation between marijuana uses and head/neck cancers was also assessed in a study involving 173 patients and 176 healthy controls. A 2.6-fold increase of occurrence of head and neck squamous cell carcinoma risk was found in marijuana users, with dose-response tendencies reported for duration and frequency of marijuana use. In fact, dose-response relationships were reported for rate of marijuana use/day and years of marijuana use. These correlations were greater for younger subjects (OR, 3.1; 95% CI, 1.0–9.7) [74]. In contrast, no association and no dose-response trends were demonstrated in a population of 407 carcinomas of the oral cavity and 615 healthy controls [75].

Finally, convincing evidence suggests that, in the testis, a correct CBs activity, associated to an appropriate CBRs signaling, is essential for spermatogenesis. Any modification of this system negatively disturbs male reproduction, from germ cell differentiation to sperm function, and might have also an effect on the onset of testicular tumors. Marijuana use has been proposed as a risk factor for testicular cancer development by augmenting the occurrence of testicular tumors. CBs, by binding to CBRs at a central level, could alter the hypothalamic–testis axis, thus disturbing normal hormone regulation of spermatogenesis and causing carcinogenesis. Moreover, CBs could bind, at the periphery in the testis, to CB receptors present in germ cells or somatic cells, modifying germ cell development and triggering the cancerous transformation. Recently, increased attention has been focused this, following the detection of epigenetic implications of cannabis exposure in germ cells [76]. In a recent report, heavy cannabis use was associated with the incidence of testicular cancer (AHR 2.57, 95% CI, 1.02, 6.50) [59].

Overall, the above data indicate that alterations of ECS expression may have a relevant effect in carcinogenesis [77,78], and these findings have led the scientific community to hypothesize that CBs may be considered as a possible therapeutic approach for the treatment of solid tumors. However, the antineoplastic potential of CB_S_ has been known for at least 50 years [55]. The oral administration of these compounds slowed the tumor progression in a mouse experimental model of Lewis lung adenocarcinoma. Similarly, Carchman et al. confirmed that CBs administration, such as D8-THC, D9-THC, and CBD, blocked both DNA synthesis and proliferation of lung adenocarcinoma in cells as well as in animal tumor models [79].

An antiproliferative effect of CBs was reported in both in vitro and in vivo models of different tumors, including pancreas, breast, and prostate cancers, but also glioma and colorectal carcinoma [80,81,82,83]. Other CBs also have anti-tumor effects, such as JWH-015, which is a naphthoyl indole derivative that induced programmed cell death in breast cancer cell lines. In addition, JWH-015 significantly decreased tumor proliferation in non-small lung cancer cell lines [83,84], while JWH-133, a pyran derivative substance, blocked tumor advancement in breast cancer cell lines [85].

However, despite the successes obtained from the in vitro and in vivo experiments, the first human interventional study to evaluate the anti-tumor effect of CBs was performed only in 2006 [64]. This was a phase I clinical trial carried out on patients with relapsed glioma. Δ9-THC intracranial administration significantly increased the median survival of treated patients [64]. Despite this, the fear of side effects has limited the experimentations for the evaluation of the antineoplastic effects of these substances. Nevertheless, CBs display a fair drug safety profile, and their possible adverse effects are within the range of those accepted for other drugs, especially in the cancer field. This new awareness and the possible synthesis of new CB1R/CB2R agonists and/or antagonists less hydrophobic and with pharmacological features that could elude the pharmacokinetic and pharmacodynamics limits of THC should ensure the safe profile for future pre-clinical studies as well as clinical trials on the topic.

## 3. CBs, Tumors, and Treatment-Related Symptoms

Several clinical studies highlight CBs effects in patients affected by hematological malignancies to evaluate if the treatment with CBs might reduce symptoms, thus ameliorating the quality of life (QOL) of patients [86]. In fact, with the progression of the diseases, several symptoms (anorexia, cachexia, fatigue, cognitive damage, anxiety, depression, neuropathic pain, sleep disorders) may appear because of malignancy or because of the treatment. Both hematologists and oncologists maintain that CBs may be considered as a therapeutic approach applicable for different tumor-related symptoms [87,88]. For instance, several studies demonstrated that THC treatment completely blocked vomiting in patients undergoing chemotherapy [89,90,91,92]. The antiemetic effect of CBs is due to modulation of both CB1R and 5-hydoxytryptamine receptors. The antiemetic advantages of THC treatment seem to be related to specific chemotherapy drugs; in fact, it has been observed that patients treated with doxorubicin and cyclophosphamide did not respond [93].

Other preclinical research suggests that CBs might be considered useful in managing the symptoms and side effects of chemotherapeutic drugs. THC decreased the cisplatin-caused emesis in a dose-dependent manner [94], confirming the results observed in other in vivo studies, which demonstrated the efficacy of Δ9-THC, Δ8-THC, Nabilone and HU210 in blocking emesis [95,96,97,98,99]. Moreover, CBs might stimulate appetite, and Dronabinol is a drug authorized for the therapy of anorexia and weight loss in adult patients with HIV, but not in tumor-related anorexia and weight loss [100]. CBs regulate appetite via the central and peripheral systems acting on limbic, hypothalamic, and intestinal areas [101]. The first study that showed endocannabinoids’ role in the control of food intake was published in 2002 and demonstrated that 2-AG levels increased in the hypothalamus and in the limbic forebrain in fasting rats [102].

CBs may also reduce neuroplastic pain; in fact, their analgesic effects, through brainstem circuit modulation, have been described in experimental studies [103,104,105,106]. Moreover, CBs treatment decreases chemotherapy-caused neuropathy, which may be considered as a further analgesic effect of CBs. For instance, the compound WIN55,212-2 was able to decrease the allodynia caused by vincristine after binding both CB1 and CB2 receptors in an in vivo experimental model [107]. However, CBs may modulate pain through the regulation of different mechanisms: THC may block prostaglandin E-2 and glutamate production, increase lipoxygenase expression, modify dopaminergic activity, reduce 5-hydroxytryptamine discharge, and stimulate TRPV2 [58,108,109,110,111]. Moreover, CBD may have anti-inflammatory effects by reducing ROS generation, pro-inflammatory cytokines, and immune cell adhesion, also alleviating pain [112,113,114].

On the other hand, although some scientific evidence has demonstrated that modulation of chronic pain in tumor patients has moderate-quality effects, most of the results were not statistically significant. Finally, other reports stated no evidence that CBs reduced pain in patients with neoplastic pain, which suggests the need for well-designed clinical trials to confirm CBs efficacy in treating chronic neoplastic pain [86,115].

## 4. Anticancer Mechanisms and Carcinogenic Actions of Cannabinoids

**Figure 1:**
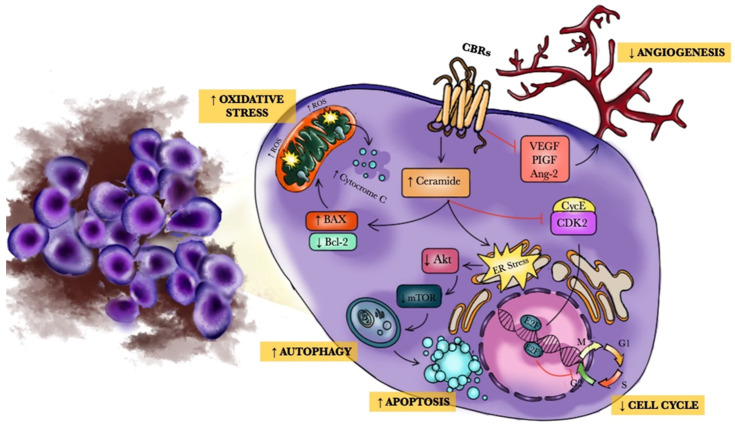
Antitumor mechanisms of CBs.CBs have a pro-apoptotic effect via an action on apoptosis regulators (Bcl-2 and BAX) as well as an effect on oxidative stress. CBs stimulate ceramide generation and cell death in tumor cells but not in normal cells, reduce angiogenesis, and exert an antiproliferative effect acting on the AKT/mTOR pathway. Finally, CBs can stimulate ER stress, thus stimulating both an AMP-activated protein kinase and the calcium/calmodulin-dependent protein kinase, kinase 2, which in turn activates autophagy. Abbreviations: CBs: Cannabinoids; BAX: bcl-2-like protein 4; Bcl-2: B-cell lymphoma 2; VEGF: vascular endothelial growth factor; Ang-2: Angiopoietin-2; PIGF: placental growth factor; AKT/mTOR: Protein kinase B/mechanistic target of rapamycin; ER: Endoplasmic reticulum.

As reported above, preclinical findings suggest that THC, CBD, and other synthetic CBs may induce tumor cell death and block tumor growth [116], with a mechanism of action that engages proliferation and apoptotic pathways, an effect on autophagy, an antiangiogenic action, on the pathways that regulate the cell cycle, on the mechanisms of immunosurveillance, and on the cells of the tumor microenvironment (Figure 1).

Of particular interest is the fact that CBs may exclusively affect neoplastic cells, while normal cells are less susceptible. Several mechanisms have been proposed to explain this effect. In some conditions, there might be a diverse stimulation of signaling pathways in cancer cells. For instance, a pathway that has been demonstrated to be differently activated by CBs in normal cells and in tumor cells is the RAS-MAPK/ERK pathway in cerebral cells. Glioma cells and normal astrocytes respond differently to THC exposure. THC stimulates ceramide generation and cell death in glioma cells but not in normal cells, which are instead safeguarded from oxidative stress by CBs. Similarly, in MCF7 breast cancer cells, data support evidence that CBD can modulate mitochondrial function and morphology in a dose-dependent manner, with clear evidence of it inducing oxidative stress at higher concentrations [117].

**Figure 2:**
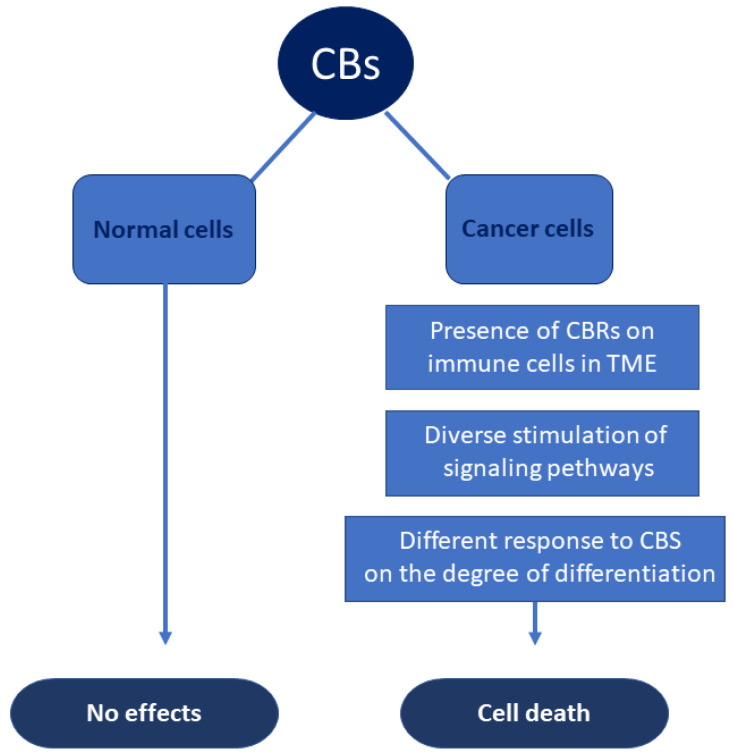
Differential effects of CBs on normal and neoplastic cells.

In other tumor cells, the different reaction to CBs may depend on the different expression of CBs receptors. CBs receptors are often greater expressed in cancer cells than in normal cells, thus increasing sensitivity to CBs in neoplastic diseases. There are also several data proposing that tumor cells may react differently to CBs depending on their condition of differentiation [118]. The different cell response may be related to CBs interaction with CB1Rs and CB2Rs or with the TRPV family, proposing that CBs may have different cell targets depending on tumor type [119]. In fact, CB2Rs and, to a lesser extent, CB1Rs are present on a multiplicity of immune cells in tumor microenvironments (TMEs). The stimulation of CBRs regulates different biological actions on cells of the adaptive and innate immune system. The expression of CB2Rs and CB1Rs on different subsets of immune cells in TME could be relevant in tumor progression [120] (Figure 2).

Regarding the antineoplastic mechanism of action of other new CBs, a study published on the cannabinoid properties of a new family of chromenopyrazoles showed that fully selective CB1R ligand lacked psychoactive effects. The main mechanism of action of quinones (PM-49 and HU-331) is ascribed to programmed cell death activation [121,122]. HU-331 is an oxidation derivative of CBD and exerted anti-cancer effects through blocking topoisomerase-II. For instance, in lymphoma and leukemia cells, PM-49 showed pro-apoptotic effect by interacting with CB1R and modulating cellular oxidative stress systems [123,124].

However, CBs may play other significant roles, thus acting on other cell processes that may increase their antitumor potential. It has been demonstrated that CBs may block angiogenesis by inhibiting the vascular endothelial growth factor (VEGF) signaling pathway. Previous studies showed that CBR agonists may reduce both VEGF production and its receptors 1 and 2 (VEGFR1, VEGFR2) in glioma, thyroid cancers, and skin tumors [125,126]. Moreover, CBs can directly reduce endothelial cell growth, which is induced by tumors [127,128]. For instance, Δ9-THC reduced both angiopoietin-2 (Ang-2) and placental growth factor (PIGF) production in tumor cells [129], whose effect in neoplasms and chronic myeloproliferative diseases is well known [130].

Endocannabinoids’ anti-cancer effects are regulated by the sophisticated modulation of different pathways. Cell-cycle arrest in G1-S phase was caused by AEA administration through the increased production of p21waf, p27 kinase inhibitor protein 1, proteolysis of Cdc25A, and reduction of the cyclin Ecyclin dependent kinase 2 kinase [131,132]. Δ9-THC blocks Ras homolog gene family member A (RHOA), focal adhesion kinase (FAK), and protein kinase Src (RHOA-FAK-Src) axis with its binding with CBRs. Moreover, the CB2R may be considered as an essential controller of the HER2 (human epidermal growth factor receptor 2) oncogene, which when increased, may contribute to an augmented susceptibility to leukemia provoked by viral infection [133]. Finally, CBs interaction with CBRs have been reported to induce autophagy in several cancer cell types [134,135,136,137].

Other mechanisms might be essential to explain the process of CB-caused cell death in some tumoral cell lines. CBs can stimulate endoplasmic reticulum (ER) stress, thus stimulating both an AMP-activated protein kinase and the calcium/calmodulin-dependent protein kinase, kinase 2, which in turn activates autophagy [136].

CBs may also play an immune-mediated action, in particular on neoplasms; in fact, CB receptor transcripts may be found in human spleen, tonsils, and peripheral blood leukocytes, with a distribution profile different among the diverse blood cell subpopulations: B cells, natural killer cells, and then polymorphonuclear neutrophils, monocytes, and T4 cells [138]. Compared with control rats, rats under morphine exposure exhibited CBR2 upregulation in the spleen and periphery blood mononuclear cells (PBMCs). IgG and IgM values in the plasma were also altered [139]. Moreover, trans-caryophyllene (TC) is a specific agonist of the CBR2. Administration of TC could inhibit the induction of vascular cell adhesion molecule-1 (VCAM-1) both in vitro and in vivo [140].

However, CBs seem able to cause both an anti-inflammatory and a suppressive effect against T lymphocytes: CBs can reduce cytokine release, and phytocannabinoids may play an immunomodulatory role both in terms of the cellular and of the humoral response, binding the CB2R [141,142]. In this context, CB2R inhibition with JTE907, a selective CB2R inverse agonist, when combined with a CB1R/CB2R gene silencing approach, demonstrated that CB2R, but not CB1R, is responsible for phytocannabinoid-mediated immune suppression [143]. CB2R modulation may decrease T cell immune responses in T cell–mediated diseases and at the same time positively may control T-independent immune responses [144].

Even if CB1R is not related to immune response modulation, it has been proposed that it has a central role in controlling cannabinoid-caused polarization of cytokine production [145]; CB1R is able to regulate interleukin-1 beta release and cyclooxygenase-2 activation, with an anti-inflammatory effect [146]. Cannabinoids may also reduce the production of other pro-inflammatory cytokines such as IL-6, IL-12, and IFN-gamma; some of these cytokines stimulate T-helper (Th) cell differentiation towards the Th1 subtype, while IL-4 and IL-5 stimulate Th2 subtype differentiation. Th1 response represents an important mechanism involved in the immune response towards tumor cells, and it is possible that inflammation, when controlled by cancer-specific Th1 cells, may block tumor onset and progression. In a Th1 milieu, the proinflammatory cytokines IL-6 and IL-1 may contribute to tumor eradication by augmenting CD4 ^+^ T cell activity [147]. Moreover, CBs are powerful IL-10 inducers, which is one of the most known anti-inflammatory cytokines that may inhibit Th1 response [148].

**Figure 3:**
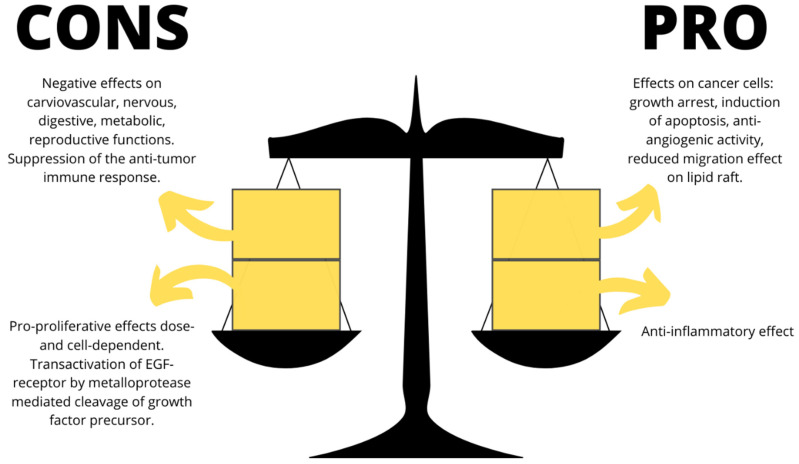
Pros and cons of the endocannabinoid system as a promising antitumor therapeutic strategy.

However, there are some reports proposing that CBs might have a tumor-promoting effect. In fact, THC, AEA, and WIN may cause the transactivation of the EGF-receptor by metalloprotease-mediated cleavage of growth factor precursors in numerous tumor cell lines, probably with a dose-dependent effect [62]. In different hemopoietic cell lines, whose growth depends on the presence of cytokines such as IL-3 or IL-6, low doses of CBs stimulated the cytokine-caused cell growth. However, this action was not due to the activation of CBs receptors but implicated a CB1/CB2 independent activation of specific signaling, such as the p42/p44 MAPK pathway. Moreover, as reported above, CBs can inhibit cell and humoral immune responses and influence the immune surveillance system; hence, these effects must be studied targeting the CBs system in vivo (Figure 3). If the tumor cells do not present CB-receptors, CBs administration, such as THC, to animals transplanted with tumors missing CB-receptors may stimulate tumor proliferation in vivo via inhibition of an efficacious anti-tumor immune response [60,118].

## 5. CBs and Hematological Malignancies

### 5.1. Preclinical Studies

Although most of the studies in the literature concern the relationships between solid neoplasms and CBs, several studies have been conducted to evaluate CBs effects on hematological malignancies. As a first approach, in vitro experimental studies have described their antitumor effects against different sorts of hematological malignancy (Table 3). For instance, CBs have been stated to be effective in cells that showed the profile of the acute lymphoblastic leukemia (ALL) and acute myeloid leukemia with lymphoid differentiation pattern [149,150].

In an experimental study in which THC was used, a powerful antileukemic efficacy in acute leukemia cell lines as well as in native leukemia blasts cultured ex vivo was demonstrated. Remarkably, an increase of programmed cell death and a reduction of proliferation mechanisms were described [129]. In an interesting experimental setting, plasma was collected from a patient treated with dronabinol under palliative supportive care and was then used as a culture medium for Jurkat cells; Dronabinol was administered as 2.5% solution and did not cause any side effects. In addition, this patient did not receive any cytoreductive therapy. A significant plasma inhibitory action was demonstrated, such that the possible antileukemic effect of Dronabinol was hypothesized [149]. In a different experiment, CP55940, a synthetic cannabinoid that mimics the effects of naturally occurring THC, also induced cell death in Jurkat cells via a CBR-independent mechanism, but mediated by a H_2_O_2_ signaling pathway. Remarkably, CP55940 showed a cytotoxic effect to ex vivo T-ALL cells obtained from chemotherapy-resistant subjects [157].

The possible CBs synergistic effect with chemotherapy may represent a promising therapeutic approach to face the chemoresistance mechanisms observed in some patients in clinical practice. For instance, Δ9- THC and CBD have demonstrated efficacy in overturning multidrug resistance in human T lymphoblastoid leukemia CEM/VLB100 cell line [143]. Moreover, encouraging results have been obtained in the field of solid tumors. Griffiths et al. evaluated the potential influence of CBD usage on therapeutic outcomes in ovarian cancer patients. As the development of chemoresistance in ovarian cancer results in treatment failure, the potential for CBD to augment the efficacy of conventional chemotherapeutic and epigenetic drugs is a topic of significant importance [141].

CBD and Δ9-THC also activated programmed cell death and reduced viability in murine lymphoma (EL-4) and human leukemia (MOLT-4) cell lines [151,152].

The combined use of CBs may also represent a fascinating therapeutic approach to further strengthen their efficacy. In an in vitro study, CBs were administered alone or in association with other CBs to enhance the positive effects against leukemia cells. Moreover, CBs such as CBD and THC were also administered with the anti-leukemia drugs cytarabine and vincristine. The results obtained from this study demonstrated that CBs could be paired together to ensure a greater effect compared to that obtained when CBs were used alone. The combined use of drugs may also adjust their therapeutic dose: it has been demonstrated that IC50 values of CBD and THC were 8 and 13 µM, respectively, when administered alone; when combined, IC50 was 4 µM. Moreover, the most effective CB pairs further synergized when administered with cytarabine and vincristine and were also able to sensitize leukemia cells to their cytotoxic actions. In addition to the differences of the IC50 doses, another important factor to consider is the sequential order of administration: CBs administration following chemotherapy caused a superior stimulation of programmed cell death [158]. Overlapping results were obtained on T-cell leukemia cell lines [159]. Discordant results were found on the interaction between CBs and CBs receptors, and it is not clear whether this interaction is specific and involves all or only some CBs receptors. For instance, cannabidiol incubation caused a CB2R-mediated decrease in cell viability in favor of programmed cell death activation in Jurkat and MOLT-4 cells and EL-4 leukemia cells [151]. However, in a different report, CBD directly affected mitochondria in T-ALL cells and modified their capacity to handle Ca^2+^, which in turn modified several cell functions. Nevertheless, in this report, pharmacological analysis demonstrated that CB1/2 receptors were not implicated in the CBD-induced [Ca^2+^]_i_ rise [153]. Both the different experimental conditions and the different cell types used in the experiments could justify the different results obtained by the authors [151,153]. These diverging results need to be addressed by further investigations. However, all studies confirmed CBs efficacy in reducing tumor cell number, either when used alone or in combination with other treatments comprising chemotherapeutics and irradiation [160,161]. Cell lines originating from acute lymphoblastic leukemia of T lineage (T-ALL), but not resting healthy T cells, are greatly responsive to CBD administration. In this case, CBD targets mitochondria and changes their ability to handle Ca^2+^. At toxic dosage, CBD causes mitochondrial Ca^2+^ surplus, constant mitochondrial transition pore formation, and consequently cell death [153]. CBD administration also determined alterations on cell morphology, ER, and Golgi, thus reducing cell size and inducing vacuolation [153]. Therefore, CBs may increase intracellular stress and modify mitochondrial membrane potential, causing cytochrome c discharge and cleavage of caspases 8, 9, 2, and 10, up to cell death [150,151,152,153,157,158,159,160,161,162,163]. Oxidative stress mechanism activation may be involved in the onset and progression of hematological malignancies; for this reason, CBs antioxidant effects might be exploited on neoplastic cells in hematological diseases [164,165,166,167]. CBD treatment for 24 h caused increased ROS concentrations in Jurkat and MOLT-4 cells, and the combined treatment with tocopherol and NAC, used as ROS scavengers, decreased CBD’s killing effects. CBD exposure also augmented the expression of the NAD(P)H oxidases NOX4 and p22phox, while blocking NOX4 and p22phox reduced ROS concentrations and diminished CBD-caused cell toxicity [151]. Coherent with these findings, ROS amounts were remarkably augmented after only two hours of CBD exposition in EL-4 cells, with a contemporaneous reduction in cellular thiols [168].

Kalenderoglou et al. studied CBD effects on the mTOR signaling in Jurkat cells [154]; CBD reduced AKT phosphorylation and ribosomal protein S6. Moreover, CBD effects were evaluated with nutrients and oxygen contents. The anti-growth effect of CBD was higher with 1% serum than with 5% serum, alone or together with doxorubicin. Cells grown in a condition with 12% of oxygen (physiological normoxia) were more resistant to CBD. Resistance to CBD under physiological normoxia would imply that CBD use might represent an issue for its anti-leukemic effect in the clinical practice. Low levels of CBD did not affect cell growth of Jurkat cells, whereas when used at high concentrations, both autophagy and the intrinsic programmed cell death pathway were triggered [153].

The effectiveness of CBs against hematological neoplastic cells was also confirmed in different lymphoid cells. Mycosis fungoides (MF) is the most frequent sort of cutaneous T-cell lymphoma (CTCL), distinguished by patches, plaques, and tumors. Sézary is a leukemic phase of CTCL, displaying with erythroderma and the occurrence of Sézary T-cells in peripheral blood [169]. A study demonstrated the cytotoxic effects of substances obtained from whole cannabis extracts on CTCL cells. The analysis was performed on My-La and HuT-78 cell lines, and active compounds were recognized in the crude extract fractions S4 and S5. Their synergistic mixture caused cell cycle arrest and programmed cell death activation [155]. Finally, non-Hodgkin lymphoma cells showed greater mRNA expression of CB1 and/or CB2 receptors compared to that observed in reactive lymphoid tissue [170], and an increased expression of CB1 and CB2 receptors was observed in mantle cell lymphoma cells with respect to normal B lymphocytes and reactive lymphoid cells [171]. AEA also decreased cell viability and induced programmed cell death in these cell lines [156].

Therefore, in vitro studies seem to confirm CBs efficacy in inducing cell death mechanisms in cancer cells and preferentially over normal cells. The studies described so far confirm that CBs use might overcome multidrug resistance and might have a synergistic effect with other chemotherapeutic agents. The intimate mechanisms and the specific signaling pathways involved still need to be better defined, since these mechanisms and pathways might be different, depending on the investigated cell types.

### 5.2. Clinical Studies

Although the therapeutic effects of cannabis have been demonstrated in several preclinical studies, few clinical studies are available.

As for the mere epidemiological aspect, two reports on non-Hodgkin’s lymphoma, which included 378 subjects [172] and 1,281 cases and 2,095 controls [173] demonstrated null to inverse correlations with marijuana use. However, parental marijuana intake in pregnant women has been correlated with childhood tumor development, including leukemia [174]. An analysis was performed to assess if lifetime cannabis intake might worsen the prognosis of 106 patients affected by chronic myelogenous leukemia or primary myelodysplastic syndrome [175]. None showed criteria for current substance dependence, but the lifetime percentage of substance use was 28% for alcohol, 12% for cannabis, and 9% for cocaine. Although lifetime cocaine use was related to a six-fold increased risk of death, neither lifetime alcohol nor cannabis intake were correlated with survival. However, some studies have attempted to evaluate the efficacy of the administration of CBs in patients affected by hematological diseases. The aforementioned idea of combining CBs with traditional chemotherapy to induce a synergistic effect seems plausible. CBD and THC can enhance the cytotoxic activity of several chemotherapeutics used for the treatment of hematological malignancies, such as cytarabine, doxorubicin, and vincristine. In particular, these drugs may act by reducing p42/44 MAPK activity, decreasing P-glycoprotein (vinblastine), blocking ABCG2 (mitoxantrone), and increasing TRPV2 channels (bortezomib, carmustine, doxorubicin) [132].

Preliminary experiments have aimed to use the combination of CBs and radiation; this approach also provided encouraging results [176]. Programmed cell death stimulation by CBD via ROS production may be responsible for DNA damage caused in tumor cells by conventional radiotherapy. Such a strategy would make it possible to decrease the number of radiation applications, thus reducing side effects in favor of therapeutic efficacy. Finally, the repercussions of cannabis in pediatric hematology are restrained to the publication of few case reports. For instance, a case study was performed on a 14-year-old male patient with a severe ALL, who was treated with a CB extract per os; this therapeutic approach did not modify the prognosis of the disease [177].

Although in vivo studies also show encouraging results in the treatment of hematological neoplastic diseases, the absence of large, controlled studies and the lack of an adequate follow-up make CBs clinical use more difficult. In particular, studies on younger patients and the evaluation of side effects, even late ones, seem urgent and indispensable.

## 6. CBs and Bone Marrow Transplantation

Despite novel prophylactic immunosuppressive therapy, graft-versus-host disease (GVHD) remains the principal reason for mortality after allogeneic hematopoietic cell transplantation (alloHCT), affecting 50% to 70% of patients receiving transplants from an HLA-matched unrelated donor [178].

In vitro results demonstrated that CBs reduce activated lymphocyte proliferation and alter cytokine production. Khuja et al. also discovered that CBD and THC utilize different receptors to mediate these effects. In vivo, in a syngeneic transplantation model, they demonstrated that all treatments inhibit lymphocyte reconstitution and showed the inhibitory role of the CB2R on lymphocyte recovery. Although pure cannabinoids exhibited a superior effect in vitro, in an allogeneic (C57BL/6 to BALB/c) BMT mouse model, THC-high and CBD-high cannabis extract treatment reduced the severity of GVHD and improved survival significantly better than pure cannabinoids [179].

In an animal experimental model, GVHD was produced by the transplantation of bone marrow cells and splenocytes from C57BL-6j to Balb-c mice. The animals were treated daily with CBD, and the administration decreased mouse mortality by reducing inflammation and damage. Evaluation of the jejunum and ileum demonstrated that CBD administration decreased the levels of C-C motif chemokine ligand (CCL) 2, CCL3, CCL5, TNF *α*, and IFN*γ*. CBD also augmented the number of type 2 cannabinoid receptors on CD4^+^ and forkhead box P3^+^ cells in the intestine, which may clarify the decrease in proinflammatory cytokines and chemokines. Antagonists of the CB2R decreased the survival rates of CBD-treated mice. Moreover, treatment with CBD did not inhibit the graft-versus-leukemia response [180].

Moreover, it was reported that both arachidonoylethanolamide (AEA) and palmitoylethanolamide (PEA) could weakly inhibit TNF-α a levels in bronchoalveolar lavage fluid of LPS-treated mice and that AEA could also inhibit neutrophil recruitment [181]. These data suggest that cannabimimetic fatty acid derivatives (CFADs) have moderate anti-inflammatory activity in the airways, though they seem to lack the ability to directly relax the airway smooth muscle.

Cannabis intake has been correlated with a reduction of both lymphocyte growth after mitogenic stimulation and cytokine levels such as IL-2, IL-10, and transforming growth factor in healthy subjects [182], and THC was reported to be effective in the prevention and therapy of GVHD in an experimental animal model [183]. TCH administration, in fact, significantly reduced liver and intestinal damage, thus increasing animal survival. Moreover, THC decreased the proliferation of donor originated effector T cells and augmented Foxp3 regulatory T cell proliferation. Like THC, CBD has powerful immunosuppressive and anti-inflammatory effects [184,185], particularly due to the modulation of T cell activity, resulting in a reduced discharge of pro-inflammatory cytokine (IL-1, IL-6, INF, TNF, and IL-17) and an increased release of anti-inflammatory cytokines, such as IL-4, IL-5, IL-10, and IL-13 [186,187]. Moreover, CBs have been reported to decrease the ability of dendritic cells to transfer to secondary lymphoid tissues and stimulate naive T cells [188].

A phase II clinical trial carried out on 48 patients (79% with acute leukemia or myelodysplastic syndrome) evaluated if CBD might reduce GVHD severity and occurrence following alloHCT [189]. GVHD prophylaxis was performed by cyclosporine and methotrexate administration. Patients receiving transplants from an unrelated donor received small dosages of anti-T-cell globulin. CBD was administered per os at the dose of 300 mg/day starting seven days before transplantation, up to day 30. None of the subjects presented acute GVHD while consuming CBD and no severe toxic effect was observed following CBD treatment. Compared with historical controls, the hazard ratio of presenting developing grades II to IV acute GVHD among patients receiving CBD plus standard GVHD prophylaxis was 0.3 (P 1/4, 0.0002). Among patients living more than 100 days, the occurrence of moderate-to-severe chronic GVHD at 12 and 18 months were 20% and 33%, respectively (clinicaltrials.gov: NCT01385124) [189]. Remarkably, the small occurrence of acute and chronic GVHD observed in patients that received CBD was analogous to the occurrence of GVHD reported in phase I/II studies on subjects treated with new drugs such as maraviroc (CCR5 antagonist), bortezomib, and vorinostat [190,191,192].

The absence of important side effects in patients undergoing allogeneic transplantation and the good prevention exercised by the administration of CBs against graft versus host disease seem to be able to favorably influence the course of these subjects. Further studies will be necessary to establish whether their administration can allow a reduction of the immunosuppressive regimen to which these patients are subjected, with the possibility of reducing important negative effects.

## 7. Future Challenges

Cannabis use in clinical practice is described to be safe in hematological adult patients, but safety in children or adolescents has not yet been confirmed. In fact, ECS plays an essential role during brain development, as CBs receptors are widely distributed in the brain in prenatal development [193]. Endocannabinoids may modify neurodevelopment by controlling neuronal migration; for instance, CB1R modulation may regulate cell growth and synaptogenesis [194]. Of particular importance could be the neurobiological effects of cannabinoid exposure during prenatal/perinatal and adolescent periods, in which the endogenous cannabinoid system plays a fundamental role in neurodevelopmental processes [195].

It has been demonstrated that CBD and THC cause neural alterations following in utero administration in an experimental zebrafish model [196]. In fact, exogenous CBs contact during embryogenesis may affect neurotransmitter systems, thus altering motor function and reproductive systems; therefore, high doses of CBs are not suggested in pediatric patients [197,198,199]. During adolescence, CB1R stimulation regulates the relationships between the prefrontal cortex, amygdala, and hippocampus [200]; in fact, CB1R-mediated effects are implicated in the control of learning, cognition, memory, and neurogenesis [201]. Therefore, it is plausible that alteration of normal ECS by exogenous THC intake may modify several brain events. In fact, long-lasting cannabis intake is correlated with mental alteration and drug dependence in adolescents [202]. Ananth et al. reported data on the effects of medical marijuana in pediatric cancer patients [203]. They identified several possible risks of marijuana use, including an effect on neurocognitive status and the onset of mental health sequelae [203]. For this reason, a thoughtful choice about the best route of administration and the different doses (depending on different stages of disease and age) will have to be made by a specialist for the use of CBs for the treatment of hematological malignancy. Non-psychoactive CBs, also considered as possible antileukemic agents, may be used to reduce toxicity and undesirable psychosomatic complications related to the use of marijuana.

Moreover, both CBs bioavailability and efficacy may depend on the route of administration; CBs have a low water-solubility, which reduces the possibility of intravenous dispensation. On the other hand, oral administration has a limit due to CBs degradation in the acidic milieu of the stomach; similarly, inhalation may affect the respiratory tract such that the use of precise dosage remains difficult. CBs administration directly into the neoplastic mass, for instance in a solid tumor, might be considered as a promising alternative approach [204]. This therapeutic approach could be used in lymphoma patients, but an unfavorable position could preclude the use of this kind of treatment. Other solutions have been proposed, such as the use of nanoparticles planned to discharge CBs only under specific situations (pH levels in the tumor milieu) [205,206].

Finally, inhalation of vaporized cannabis may possibly expose patients to critical pulmonary infections [207,208]. However, long-acting oral formulations are the backbone of therapy for chronic conditions, while vaporization might be used as an add-on therapy for acute symptoms. The daily dose-equivalent of THC should usually be limited to 30 mg/day, in combination with CBD, if possible, in order to reduce THC side effects [209,210]. Patients generally assume CBD with the smallest quantity of THC to ameliorate their QOL while minimizing side effects.

Finally, the different content of CBs might be considered in the natural cannabis plant (3–4%) compared to the new super plants. For instance, THC (the main psychoactive compound) level is different in Dutch cannabis, commonly called “nederwiet” [211]. The average THC level of Dutch home-grown marijuana (20.4% THC) was significantly higher than that of imported marijuana (7.0% THC) [212]. Therefore, the use of this plant will have different effects in terms of dependence and immune response as well as on the relationship between CBs and neoplasms. Similar considerations should be made for the new synthetic preparations.

## 8. Conclusions

The use of medical cannabis is acquiring larger medical approval worldwide. In the field of hematologic diseases and in cancers, most CB effects, mainly directed against CB2R, are encouraging, as reduction of cell growth and increased programmed cell death have been reported [213]. In particular, CBs efficacy has been demonstrated for both acute and chronic lymphoid and myeloid diseases in the management of numerous symptoms and in the prevention of graft versus host disease after allogeneic transplantation. However, the encouraging in vitro and in vivo experimental findings have not been translated into adequate clinical trials, in spite of the rising interest in these substances. Such findings are intensely suggested to be verified with a focus on cannabinoid ligand bioavailability and cannabinoid psychotropic characteristics. The initial antineoplastic actions and their virtual absence of relevant collateral effects must be proven in animal models and in controlled clinical studies performed in large populations of subjects. Only a careful and in-depth experimental analysis will make the modulation of the endocannabinoid system a promising therapeutic target for the therapy of hematological malignancies.

For hematologists, issues relating to the possible risks and potential advantages for the management of hematological malignancies must be explained before using medical cannabis as an anti-cancer treatment. Clarifying these data would be an indispensable phase in the clinical translation of these stimulating results.

## Figures and Tables

**Table 1:** Adverse effects of marijuana intake reported in clinical trials.

Apparatus	Effect	Number of Patients	Exposure	Ref.
Respiratory tract	Chronic obstructive lung disease	878	More than 50 cigarettes	[28]
	Pneumothorax	3	Daily	[29]
	Emphysema	399	Dose-response	[30]
	Large lung bullae	4	High exposure	[31]
	Bullous lung bullae	10	Regular chronic exposure	[32]
Cardiovascular system	Hypertension, Tachyarrythmia	1	Infrequent use	[33]
	Atrial fibrillation	6		[34]
Liver	Fibrosis	204	Daily	[35]
Cognitive alteration	Cognitive defect	102	Long-term use	[36]
	Executive function	55	3 times/week	[37]
Dependence	Cognitive deficiency, psychoses, and depressive alterations	2152	Frequent or heavy use	[38]

**Table 2:** Carcinogenic potential of Cannabis and CBs reported in clinical trials.

	Apparatus	Number of Patients	Exposure	Ref.
Cancer	Lung	49,321	More than 50 times	[45]
	Head and Neck	6	Habitual use	[46]
	Transitional cell carcinoma	52	Habitual use	[47]
	Glioma	133,811	Once a month	[57]
	Head and Neck	173	Dose-response	[58]
	Testicular germ cell tumors	49,343	More than 50 times	[59]

**Table 3:** Effects of CBs in preclinical studies on hematological malignancies.

Diseases and Cells	Study	Mechanism	Ref.
Acute lymphoblastic leukemia (MOLM-13, Jurkat cells)	In vitro	Apoptosis	[149]
Acute Myeloid leukemia with lymphoid differentiation pattern	In vitroEx vivo	H_2_O_2_ mediated mechanism	[150]
Lymphoma (EL-4 cells)	In vitro	Oxidative stress	[151]
Acute promyelocytic leukemia (CEM cells, HL60 cells), Erythroblastic leukemia (HEL-92)	In vitro	Apoptosis	[152]
B-ALL (RS;11, Reh cells), T-ALL (MOLT-3 cells, Jurkat cells), Chronic myeloid leukemia (K562 cells)	In vitro	Mitochondria changes, endoplasmic reticulum stress	[153]
T cell leukemia (Jurkat cells)	In vitro	AKT phosphorylation	[154]
Cutaneous T cell lymphoma (My-La and HuT-70 cells)	In vitro	Apoptosis	[155]
Mantle cell lymphoma cells	In vitro	Apoptosis	[156]
